# Narrowband Internet of Things (NB-IoT): From Physical (PHY) and Media Access Control (MAC) Layers Perspectives

**DOI:** 10.3390/s19112613

**Published:** 2019-06-08

**Authors:** Collins Burton Mwakwata, Hassan Malik, Muhammad Mahtab Alam, Yannick Le Moullec, Sven Parand, Shahid Mumtaz

**Affiliations:** 1 Thomas Johann Seebeck Department of Electronics, Tallinn University of Technology (TalTech), Ehitajate tee-5, 19086 Tallinn, Estonia; hassan.malik@taltech.ee (H.M.); muhammad.alam@taltech.ee (M.M.A.); yannick.lemoullec@taltech.ee (Y.L.M.); 2 Telia Estonia Ltd., 10616 Tallinn, Estonia; sven.parand@telia.ee; 3 Instituto de Telecomunicações, 1049-001 Aveiro, Portugal; smumtaz@av.it.pt

**Keywords:** narrowband, IoT, PHY, NB-IoT, MAC, deployment, survey, mMTC, 5G

## Abstract

Narrowband internet of things (NB-IoT) is a recent cellular radio access technology based on Long-Term Evolution (LTE) introduced by Third-Generation Partnership Project (3GPP) for Low-Power Wide-Area Networks (LPWAN). The main aim of NB-IoT is to support massive machine-type communication (mMTC) and enable low-power, low-cost, and low-data-rate communication. NB-IoT is based on LTE design with some changes to meet the mMTC requirements. For example, in the physical (PHY) layer only single-antenna and low-order modulations are supported, and in the Medium Access Control (MAC) layers only one physical resource block is allocated for resource scheduling. The aim of this survey is to provide a comprehensive overview of the design changes brought in the NB-IoT standardization along with the detailed research developments from the perspectives of Physical and MAC layers. The survey also includes an overview of Evolved Packet Core (EPC) changes to support the Service Capability Exposure Function (SCEF) to manage both IP and non-IP data packets through Control Plane (CP) and User Plane (UP), the possible deployment scenarios of NB-IoT in future Heterogeneous Wireless Networks (HetNet). Finally, existing and emerging research challenges in this direction are presented to motivate future research activities.

## 1. Introduction

According to Information Handling Services (IHS) technology forecast, the Internet of Things (IoT) market is expected to grow to billions of devices by 2020 [1]. Massive connections are expected to respond to different IoT use cases such as smart city, smart wearables, smart home, etc. [2]. For these applications, latency-insensitive devices can be positioned in hard-to-reach areas and do not require high throughput or frequent reporting. Therefore, to cope with such tremendous IoT trends, the Third-Generation Partnership Project (3GPP) introduced the Narrowband Internet of Things (NB-IoT) standard as a communication technology enabler. NB-IoT is categorized as one of the licensed Low-Power Wide-Area Networks (LPWAN) cellular technologies based on Long-Term Evolution (LTE) with long range and low cost. In the LPWAN category, there exist other licensed technologies, i.e., Long-Term Evolution Category M1 (LTE-M), and unlicensed technologies, i.e., Long Range (LoRa), SigFox, Ingenu, etc. [3,4,5,6,7], but they are not the focus of the current work since they are not based on cellular technology.

The term Narrowband refers to NB-IoT’s bandwidth of maximum 200 kHz thanks to which it can coexist either in the Global System for Mobile Communications (GSM) spectrum or by occupying one of the legacy LTE Physical Resource Blocks (PRBs) as in-band or as guard-band. Since it coexists in the LTE spectrum, NB-IoT follows the legacy LTE numerologies as it uses Orthogonal Frequency Division Multiplexing (OFDM) and Single-Carrier Frequency Division Multiple Access (SC-FDMA) in the downlink and uplink transmission schemes, respectively. Some modifications in the physical (PHY) and medium access control (MAC) layers are implemented to support the long-range massive machine-type (mMTC) connections with low power, low data rates, low complexity, and hence low cost. However, despite its low complexity, this new radio access technology (RAT) delivers better performance in terms of the supported number of devices, and coverage enhancements for latency-insensitive applications with maximum coupling loss (MCL) of about 20 dB higher than LTE (i.e., 164 dB) [5,6,7,8,9,10,11].

**Figure 1:**
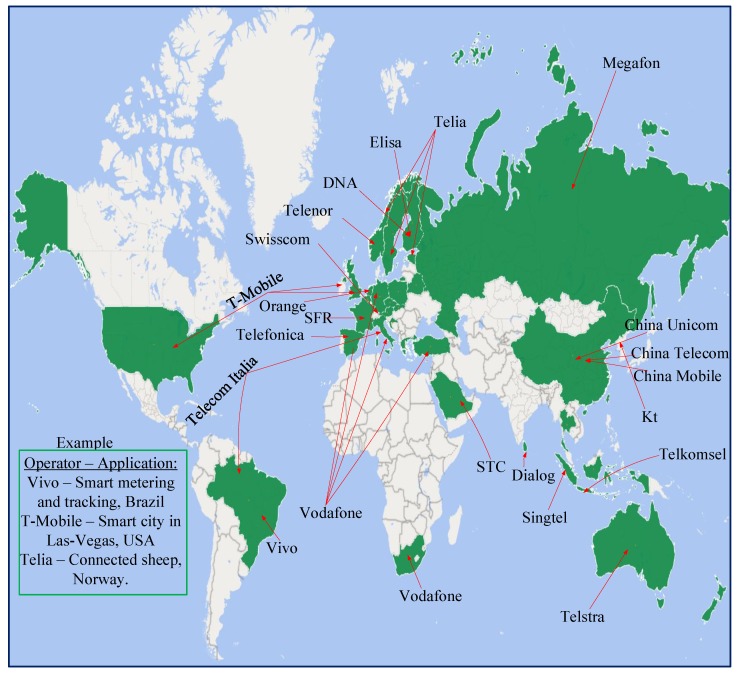
The geographical representation of countries with the ongoing NB-IoT real-life deployments for diverse use cases (May 2019).

With flexible deployment as well as the possibility to implement over-the-air (OTA) firmware upgrades, many telecommunication operators across the globe (as shown in Figure 1) deployed NB-IoT to test its practical feasibility on diverse use cases with real-life trials such as connected sheep in Norway [12], smart metering and tracking in Brazil [13], NB-IoT at sea in Norway [14], smart city in Las Vegas, USA [15], etc. The trials are enabled by different NB-IoT software and hardware solutions from different chip or module vendors such as Skyworks [16], Media tek [17], Neul (Huawei) [18], Quectel [19], Nordic Semiconductors [20], Intel [21], Sequans [22], Qualcomm [23], Siera wireless [24], Samsung [25], Altair [26], U-Blox [27], and so on.

The availability of such commercial off-the-shelf solutions speeds up the adoption of NB-IoT. For this reason, numerous studies addressing segmented enhancement criteria including survey articles emerged to analyze NB-IoT performance and implementation. Table 1 presents, in a nutshell, the main differences and similarities between this survey and the other existing ones by displaying the key focus features.

For example, in [32], the authors surveyed the development path of MTC and elaborated the NB-IoT evolution in Release 13. Similarly, in [28], the authors discussed the Release 13 features and compared its performance with respect to other communication technologies such as LTE-M, SigFox, Lora and Wireless-Fidelity (WiFi), etc. In [29,30], the authors gave an overview of NB-IoT Release 14; however, in [30], the authors elaborated more on the expectations for NB-IoT Release 15 agenda. In [31], the authors presented a survey on the NB-IoT downlink scheduling issues by highlighting the associated scheduling process in terms of offset index selection. In [33], the authors surveyed the uplink and downlink performance evaluation of NB-IoT systems by analyzing the main causes of latency, trade-off between throughput and free resources, channel occupancy etc. with respect to Release 13 and Release 14 updates.

In contrast to the above surveys, this paper presents:

- A comprehensive survey of NB-IoT, from Release 13 to the ongoing Release 16 prospects.
- An all-inclusive overview of the state of the art of PHY and MAC layers by addressing the key improvement concerns in terms of challenges and the corresponding potential solutions.
- The possible NB-IoT deployment strategies for synchronous and asynchronous network structures in HetNet scenarios to foster the NB-IoT coexistence with legacy technologies as well as with the fifth generation (5G) networks.
- Discussion on the open research challenges to motivate future research directions.

To the best of the authors’ knowledge, this is the first survey that covers broadly these above-mentioned contributions and hence will facilitate the reader’s knowledge related to NB-IoT from standardization, ongoing research, and its practical implementation.

The rest of this paper is organized as follows: Section 2 discusses NB-IoT standards by elaborating the key design changes and the related ongoing enhancements. Section 3 presents the state of the art of NB-IoT protocol stack by detailing the PHY layer and MAC layer features. Section 4 discusses the open research questions and their potential solutions, and the conclusion is drawn in Section 5.

## 2. Narrowband-IoT Standard and Releases

Early in 2014, the LPWAN market rapidly developed thanks to the emergence of IoT. Realizing the need and potential for new communication ways, 3GPP started a feasibility study on cellular system support for an ultra-low complexity and low throughput IoT solution referred to as cellular IoT. In May 2014, Huawei and Vodafone proposed the Narrowband Machine to Machine (NB-M2M) to 3GPP as a study item to cope with the IoT market needs. Additional telecom industrial players got interested and later the same year Qualcomm proposed narrowband orthogonal frequency division multiplexing (NB-OFDM). In May 2015, 3GPP merged the two proposals (i.e., NB-M2M and NB-OFDM) and formed the Narrowband Cellular IoT (NB-CIoT). Eight months later, Ericsson proposed the Narrowband Long-Term Evolution NB-LTE. In September 2015, 3GPP included all proposals as a work item for Release 13. The key difference between NB-CIoT and NB-LTE was the number of the reused legacy LTE network resources to support interoperability. In June 2016 NB-IoT was recognized as a new clean slate RAT. Only further improvement changes were allowed and implemented thereafter.

In this regard, this section presents the main NB-IoT design changes from Release 13 until today that enabled the massive IoT connections with the corresponding solutions to respond to the adopted NB-IoT objectives. The enhancement features are classified following the objectives that are presented in the releases which would make it easier for the readers to refer back to the official 3GPP documents [8,9,34,35,36,37,38].

### 2.1. Release 13

3GPP introduced the following techniques in NB-IoT Release 13 to enable cellular massive IoT deployment for diverse use cases with low power, low complexity, and hence low cost. The introduced features and their corresponding objectives are as follows.

#### 2.1.1. Mode of Operation

**Figure 2:**
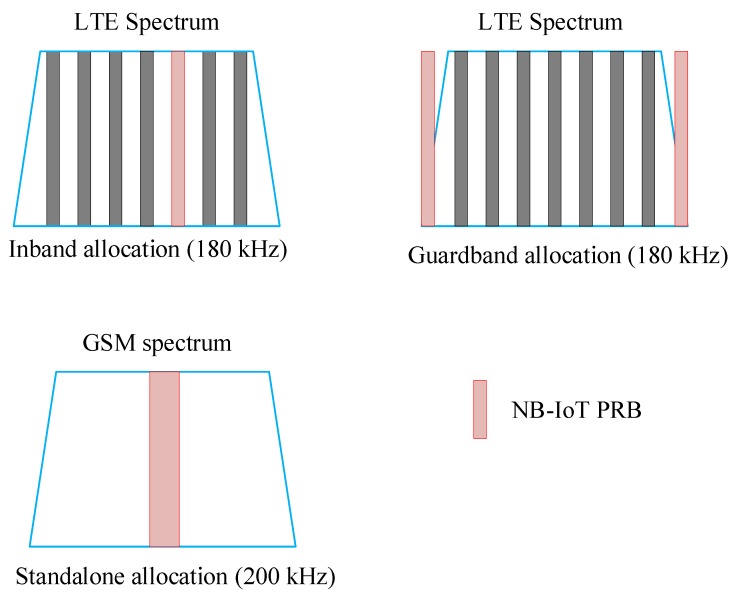
Narrow band Interet of Things (NB-IoT) Flexible Allocation inside Long-Term Evolution (LTE) spectrum (in-band and guard-band) and when refarming the Global System for Mobile Communications (GSM) spectrum (standalone).

With the limited bandwidth requirement, NB-IoT can be deployed in three different modes i.e., standalone, in-band, and guard-band, as depicted in Figure 2. In in-band and guard-band modes, NB-IoT occupies one PRBs of 180 KHz in LTE spectrum both in the downlink and uplink. It can also be allocated as standalone where it occupies the 200 KHz bandwidth by “refarming” the GSM spectrum. These flexible deployment possibilities enable fast integration and coexistence with legacy LTE and GSM systems.

#### 2.1.2. Multi-Tone Transmission Support

To reach the massive device deployment objective, NB-IoT introduces the allocation of Resource Units (RU) to multiple User Equipment (UE) contrary to LTE where the whole resource block is allocated to a single UE in the uplink. In this regard, tones (frequency domain) with different duration are allocated to UEs. For the uplink transmission, each tone may either occupy 3.75 kHz or 15 kHz of transmission bandwidth based on the SC-FDMA scheme; for downlink NB-IoT uses 15 kHz of transmission bandwidth with OFDM scheme as LTE. With 15 kHz spacing, NB-IoT can dedicate either single-tone (8 ms) or multi-tone (3 tones, 6 tones, and 12 tones) to different UEs with the duration of 4 ms, 2 ms, and 1 ms, respectively. On the other hand, the 3.75 kHz spacing supports only single-tone allocation to different users with 48 subcariers of 32 ms duration [11,39,40].

#### 2.1.3. Complexity and Cost Reduction Techniques

NB-IoT is required to have low complexity to reach the low-cost objective to facilitate massive connections. The features that were implemented to reach this objective include relaxed base-band processing, low memory storage, and reduced radio-frequency (RF) components. In this regard, the system bandwidth is set as narrow as 180 kHz with reduced frequency and time synchronization requirement. Also, NB-IoT uses the restricted BPSK and QPSK modulation schemes with only one antenna support both in uplink and downlink transmission.

#### 2.1.4. Power Reduction Method

NB-IoT devices are intended to have a 10 years battery life to support massive deployment with limited human intervention. In this regard, two features i.e., Power Saving Mode (PSM), (from Release 12), and extended Discontinuous Reception (eDRx) (new feature from Release 13) were supported. These features are intended to extend the UE’s battery longevity as follows:

In PSM, the NB-IoT device is configured to completely sleep while remaining registered online but cannot be reached by the base station signaling. In Release 13, the device can be in PSM mode for approximately up to about 413 days. In eDRX, the device is in an inactive mode for a few minutes to a few hours only.

In both cases, the partial or complete inability to receiving and sending different signals enhance the battery life longevity; however, choosing either PSM, eDRX or both depends on the corresponding use-case requirement. In this regard, the device can be synchronized to wake up from these modes by either Real-Time Clock (RTC), triggering from sensors, or both.

#### 2.1.5. Physical Channels and Signals

NB-IoT adopts the same frame structure as LTE, with 1024 hyper frames, consisting of 1024 frames that contain 10 subframes of two slots with a duration of 0.5 ms each in the time domain. Similarly, in the frequency domain, NB-IoT contains 12 subcarriers of 7 OFDM symbols mapped in each slot. In addition to that, when NB-IoT uses the 3.75 kHz spacing on the uplink, 48 subcarriers are used with a slot duration of 2 ms.

The following channels and signals are used in the uplink:

- Narrowband Physical Random Access Channel (NPRACH).
- Narrowband Physical Uplink Shared Channel (NPUSCH).
- Demodulation Reference Signal (DMRS).

And the following are in the downlink frame:

- Narrowband Physical Downlink Shared Channel (NPDSCH).
- Narrowband Physical Downlink Control Channel (NPDCCH).
- Narrowband Reference Signal (NRS).
- Narrowband Primary Synchronization Signal (NPSS).
- Narrowband Secondary Synchronization Signal (NSSS).
- Narrowband Physical Broadcast Channel (NPBCH).

In general, NPRACH is used by UEs to perform initial access to the network, to request transmission resources, and to reconnect to the base station after a link failure. NPDSCH and NPUSCH are used to carry the downlink and uplink data packets transmissions, respectively. DMRS is used for uplink channel estimation accuracy. The UE acquires Master Information Block (MIB) from NPBCH and System Information Block (SIBs) from the NPDCCH. The defined MIB and SIB are broadcasted once during 640 ms and 2560 ms intervals, respectively. The timing of the remaining SIBs is configured in SIB1-NB. NRS is used for cell search and initial system acquisition. NPSS and NSSS are used by the UE for its frequency and timing synchronization with the base station. Due to overhead scheduling gaps in NPDCCH, the downlink and uplink peak data rates are ~250 kb/s and ~2267 kb/s, respectively, [34,40,41,42,43].

#### 2.1.6. Coverage Enhancement Method

NB-IoT is designed to enhance coverage for the applications that are in hard-to-reach areas such as deep indoors and basements. In this regard, NB-IoT delivers an additional coverage of 20 dB as compared to the legacy LTE system. This corresponds to 164 dB of MCL. To enhance its coverage, NB-IoT uses up to 128 and 2048 retransmissions in uplink and downlink, respectively. Hence, this makes NB-IoT suitable for use cases that are latency insensitive as it can tolerate up to 10 seconds transmission delay.

### 2.2. Release 14 Enhancements

After the implementation of Release 13 features, studies erupted along with field trials that revealed the need for further enhancements to improve the quality of service as well as user experience. In this regard, 3GPP introduced further enhancement features to NB-IoT.

The enhancements features in Release 14 include positioning update, multicast services, and a new UE output power class in which the NB-IoT system throughput, mobility, service continuity and non-anchor carrier operation are improved [29,30].

#### 2.2.1. Improved Positioning Technique

3GPP Release 14 introduces an indoor advanced positioning method of observed time difference of arrival (OTDOA) for NB-IoT to enhance UE position measurement of cell identity (CID). In OTDOA method, the UE measures the times of arrival (ToAs) of positioning reference signals (PRSs) received from different transmitters with respect to a reference node’s PRS transmission to form the reference signal time difference (RSTD) measurements. In enhanced CID, the measurement requirements include the base station receive (Rx) and transmit (Tx) time difference, reference signal received power (RSRP), and reference signal received quality (RSRQ).

#### 2.2.2. Multicast Services

The main objective of this mechanism is to optimize resources as well as transmission latency by addressing the data to a group of UEs at the same time rather than sending it multiple times to separate devices. Therefore in Release 14, Multimedia Broadcast Multicast Services (MBMS) is supported through single-cell point-to-multipoint (SC-PTM). In general, SC-PTM is an efficient dynamic mechanism for optimal radio resource usage as it allows broadcast or multicast services to a specific group based on real-time traffic load and user requirement. SC-PTM uses NPDSCH by mapping Single-cell MBMS Control CHannel (SC-MCCH) and Single-Cell MBMS Traffic CHannel (SC-MTCH) that carry control and data traffic to the physical layer scheduled by using the downlink control information (DCI).

#### 2.2.3. New Power Class for Narrowband-IoT User Equipment

Instead of the two power classes of Release 13 (i.e., 20 dBm and 23 dBm), in Release 14, the maximum allowed device’s output power is reduced to 14 dBm. This has led to coverage relaxation of 9 dB that corresponds to 155 dB MCL as compared to 164 dB MCL and hence reduces the drained current. Technically, the use of the new power class facilitates the use of small coin-cell batteries and hence can be suitable for limited-size devices and applications that need a small battery. The compensation of the reduced NB-IoT power is achieved by increasing the NB-IoT transmission time to maintain the same energy per bit as the UE in Release 13 achieves. The newly introduced power class allows the serving base station to acquire the device power class during the establishment of the connection.

#### 2.2.4. New Transport-Block-Size Support

Contrary to Release 13 where NB-IoT supports relatively low data rates (~250 kb/s and ~226.7 kb/s in downlink and uplink, respectively), 3GPP Release 14 introduces a new NB-IoT device category which supports the improved data rates by enhancing the Transport Block Size (TBS) to 2536 bits. These data rates can be reached thanks to the ability to support a second Hybrid Automatic Repeat Request (HARQ) process. This second HARQ is useful for enhancing the reliability of the link for the UEs that experience favorable channel conditions. Implementation of this optional second HARQ process results in throughput gain as it reduces the overhead caused by NPDCCH scheduling gaps.

#### 2.2.5. Multicarrier Operation

To enable the massive NB-IoT deployment, in Release 14, NB-IoT can monitor paging and perform random access on non-anchor carriers. With this feature, one or more non-anchor carriers are added to the anchor carrier to carry out the synchronization and mobility measurements by using the NRS. Non-anchor carriers should also perform random access or paging when needed. Therefore, paging occasions and hence paging load will be spread over the anchor and non-anchor carriers and all carriers can then monitor paging.

#### 2.2.6. User Equipment Mobility Enhancement

For the use cases that involve mobility, the temporary loss of radio interface impacts the system to a degree that can degrade link performance in terms of transmission errors. In this regard, 3GPP Release 14 introduces the possibility of Radio Resource Control (RRC) re-establishment for NB-IoT UE that supports data transfer via the control plane, i.e., the UE will try to re-establish the connection on that cell and resume the data transfer. This new RRC re-establishment feature hides the temporary loss of the radio interface to the upper layers.

### 2.3. Release 15 Enhancements

On top of all the enhancements that were introduced in Releases 13 and 14, the following improvements were introduced in Release 15 to satisfy the fast adoption of massive deployment with further improved quality of service.

#### 2.3.1. Latency Reduction

In Release 15, NB-IoT supports new features to further reduce the transmission delay as well as to further reduce the power consumption dissipated during long transmission requirements. In this regard, the NB-IoT UE is now able to support the physical layer Scheduling Request (SR) which is a special physical layer message to request the network to send the access grant (DCI format 0) so that the UE can transmit the uplink data. Also, NB-IoT uses a wake-up (Wu) signal to wake up the main receiver. This signal is transmitted in idle mode only when the UE is required to decode the physical downlink control channel in paging occasions. Therefore, power consumption reduction with the wake-up signal technique is larger when the UE wakes up from deep sleep more frequently (i.e., for shorter DRX/eDRX cycles). Also, significant power consumption reduction is achieved even when a common wake-up signal is used for a group of UEs. Quick RRC release and early data transmission during random access channel (RACH) procedure are supported to reduce the UE transmission latency and hence power consumption.

#### 2.3.2. Semi-Persistent Scheduling

To enable better support of voice messages for the corresponding use cases, in Release 15, Semi-Persistent Scheduling (SPS) feature is introduced. In general, SPS is comprised of persistent scheduling for initial transmissions and dynamic scheduling for retransmissions. The base station assigns specific resource units to be used for NB-IoT UE voice messages with specific interval to save control plane overhead and hence optimize the radio resource usage. By principle, the base station preconfigures the UE with the Radio Network Temporary Identifier (SPS-RNTI) which is used to specifically differentiate one NB-IoT UE from another, or one radio channel from another. This SPS enables the NB-IoT data reception at a regular configured periodicity.

#### 2.3.3. Small Cell Support

To further improve the capacity as well as coverage, in Release 15, NB-IoT supports small cell deployments. The downlink power to be reused for NB-IoT small cells is specified in section 16.2.2 of TS 36.213 [44]. In general, NB-IoT UE is not allowed to transmit more power than the configured maximum power, even if the configured power is lower than UE’s maximum capability. This is done to avoid interference.

On the other hand, to extend the IoT connectivity especially in remote and rural areas for use cases such as agriculture, logistics, and environmental monitoring, NB-IoT is now able to support up to 100 km range. According to Ericsson, this could be achieved with a software upgrade only, without any changes in the existing NB-IoT hardware [45].

#### 2.3.4. Enhanced User Equipment Measurements

Like in legacy LTE systems, UE measurements are critical since the corresponding reporting is mainly used to characterize the reference signal of a given bandwidth.

In Release 15, UE measurements are improved in a way that only NSSS additionally to NRS is defined for radio resource management measurement enhancement. This means that NRS is determined by the resource elements that carry NSSS in the NSSS occasions that the UE measures, through which the cell search and initial cell acquisition are improved.

#### 2.3.5. Time Division Duplex (TDD) Support

In Release 15, a new feature of TDD support is introduced with a new TDD frame structure (type 2). For both 3.75 kHz and 15 kHz spacing, some specified restrictions are introduced i.e., only a normal cyclic prefix is supported for NB-IoT transmission. To support some of the TDD configurations with few downlink subframes, some of the system information (SI) can be transmitted on non-anchor carriers. In this way, the UE will have reduced system information acquisition and search time, and hence reduced UE differentiation and access control [30,46,47].

### 2.4. Release 16 Enhancement Prospects

3GPP and many industrial players are involved in ongoing discussions for Release 16 enhancements. The agenda includes the following objectives with their corresponding solutions.

#### 2.4.1. Grant-Free Access

Most of the power consumption takes place during the NB-IoT UE active time, i.e., during Tx and Rx. In Release 16, the UE will be expected to transmit during RRC-Idle mode through Msg3 (RRC connection request) without access grant. A UE in RRC connected mode can transmit data without grant or with the simplified control-less grant. A further enhancement is on reducing NB-IoT signaling overhead while guaranteeing the needed quality of service. These features will reduce both power consumption and latency. In Release 16, it is also proposed to further study other signal waveforms (i.e., FDMA) that require less orthogonality with more relaxed timing advance (TA) alignment as compared to SC-FDMA.

#### 2.4.2. Simultaneous Multi-User Transmission

The introduction of new schemes will enable simultaneous multi-user transmissions by using a shared resource in the time and frequency domains, such as Code division multiplexing (CDM), and multi-user multiple inputs multiple outputs (MU-MIMO), without increasing the number of antennae at the UE. In this regard, more dynamic access can also be achieved through enhanced base station receiver for detection of multiple users that are using the same resource unit as cluster and hence be able to schedule them effectively. This is because, for the last releases, NB-IoT UE uses the static or semi-static configuration of more resources for the unexpected application traffic handling. Similarly, the introduction of NB-IoT transmission without grant will cause a collision of data packets so dynamic handling of multiplexing is necessary.

#### 2.4.3. Enhanced Group Message Mechanism

In Release 16, there should be more enhancements to support downlink command between user groups and group RNTIs. This is because MBMS which was proposed in Release 14 is only efficient for large size downlink command message transmission and requires many UEs to be deployed. For example, the application layer common message can be very small but sent to many UEs under a small group of UEs hence making MBMS not efficient for such applications.

#### 2.4.4. Inter-RAT Idle-Mode Mobility

For applications such as smart tracking of logistics that involve mobility, the NB-IoT UE may still need to be accessible even when moved to the area served by other base station.

In this regard, 3GPP should introduce the new feature for NB-IoT UE support for inter-RAT mobility during idle mode. The mentioned feature is introduced along with optional handover support during connected mode through procedure simplification i.e., without dedicated signaling for measurement control and report. This is because handover helps to reduce system information reading time.

#### 2.4.5. Network Management Tool Enhancement to Improve UE Differentiation

NB-IoT UE is expected to be able to perform differentiation according to maximal tolerable delay per service to optimize the radio resource usage. This is because, in the last release, the UE can be differentiated according to traffic model (periodic communication indicator, periodic time, scheduled communication time, traffic profile) and battery indication.

Section 2 has presented the NB-IoT standard and the corresponding enhancements from Release 13 until today. It has highlighted the main design changes and the corresponding further enhancements, i.e., deployment flexibility, physical channels and signals, positioning, multicast, new power classes, improved data rates, multicarrier operations, mobility support, improved scheduling, NB-IoT small cell support etc.

## 3. Narrowband-IoT: Protocol Stack

This section presents the NB-IoT protocol stack based on state of the art of the PHY and MAC layers to identify the knowledge gap and define future research directions. NB-IoT adopts the same protocol stack as the legacy LTE. However, some design changes in both PHY and MAC layers were introduced to support the massive long-range connections with up to additional 20 dB MCL than in legacy technologies such as LTE, GSM, and GPRS. Those changes are described in what follows.

### 3.1. Physical Layer

**Figure 3:**
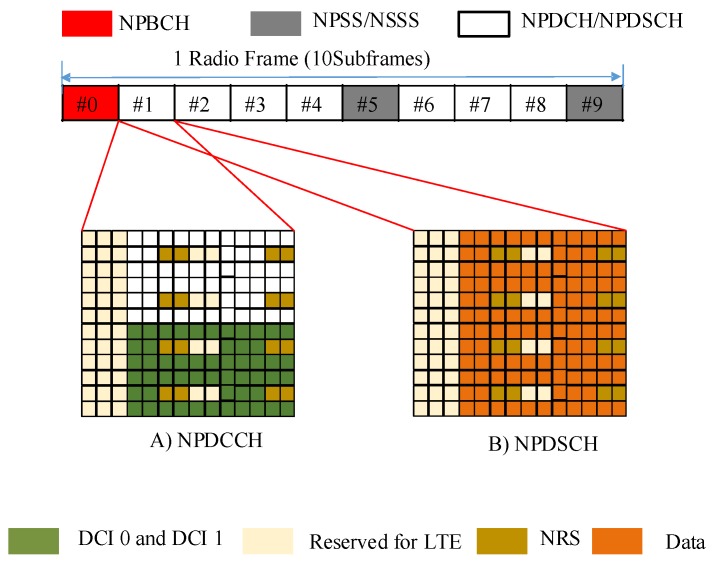
NB-IoT Downlink Frame Structure: subframe number 0 carries the Narrowband Physical Broadcast Channel (NPBCH), 1 to 4, and 6 to 8 carry the Narrowband Physical Downlink Control Channel (NPDCCH)/Narrowband Physical Downlink Shared Channel (NPDSCH), and 5 and 9 carry the Narrowband Primary Synchronization Signal (NPSS)/Narrowband Secondary Synchronization Signal (NSSS) (**A**) When the subframe is carrying control channels and (**B**) when the subframe is carrying data.

**Figure 4:**
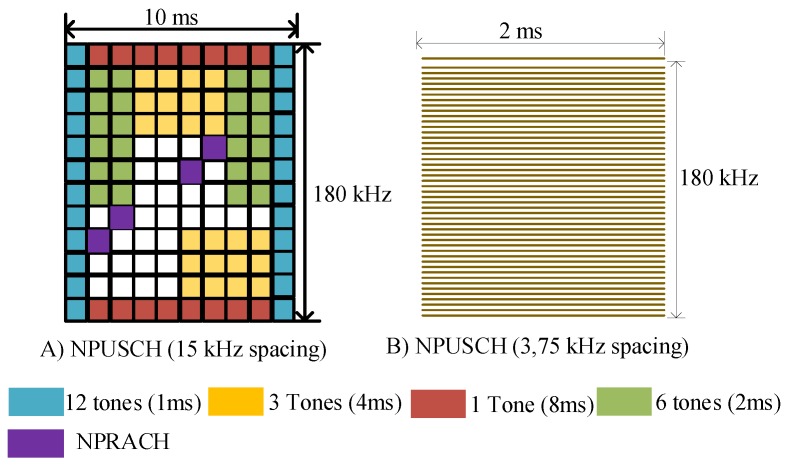
NB-IoT Uplink Frame Structure, (**A**) when 15 kHz spacing is used with different tone-allocation possibilities with slot duration of 0.5 ms and (**B**) when 3.75 kHz is used only single-tone allocation is supported with 4 times longer slot duration (2 ms).

On the physical layer, NB-IoT adopts the same numerologies as legacy LTE along with OFDM and SC-FDMA signal waveforms in downlink and uplink, respectively. However, the resource scheduling unit in NB-IoT is the subcarrier (or tone) instead of PRB, to foster the network scalability by serving multiple UEs in a 180 kHz bandwidth. The downlink and uplink frame structures are as depicted in Figure 3 and Figure 4, respectively.

In general, the base station uses DCI to specify the scheduling information for a downlink/uplink transmission in NB-IoT. Then NB-IoT UE learns the deployment mode (standalone, in-band, or guard-band) as well as the cell identity through its initial acquisition, and it figures out which resource elements are already used by LTE. This is the way by which the UE can map NPDCCH and NPDSCH symbols to available resource elements. For example, in the downlink, NPDCCH is transmitted by aggregating the narrowband control elements (element 0 and element 1) where element 0 is occupied in subcarrier 0 to 5 and element 1 occupies subcarrier 6 to 11 in a subframe. The elements are determined by the type of DCI which is carried by NPDCCH to deliver scheduling command. Either two DCIs can be multiplexed in one subframe, or one DCI can be mapped in one subframe, corresponding to the aggregation level used [48]. However, NPDCCH, NPDSCH, and NRS cannot be mapped to the already occupied resource elements for LTE signals such as cell-specific reference symbols (CRS) and LTE physical downlink control channel (PDCCH). When NB-IoT UE receives NPDCCH which carries DCI, it decodes it and uses the device’s scheduling feature (k0) to know the delay over which it will start to receive NPDSCH. The scheduling information is used to identify the allocated resources over NPDSCH and NPUSCH, respectively. In each NPDCCH, a maximum of two DCIs can be transported, and each UE can receive up to one DCI. The time interval between two successive NPDCCH opportunities is referred to as an NPDCCH period (PP) [48].

In the state of the art, different works have proposed solutions to the challenges that occur in PHY layer features, such as initial cell acquisition and synchronization, random access, channel estimation, error correction, and co-channel interference, as summarized in Table 2.

#### 3.1.1. Cell Acquisition and Synchronization

NB-IoT UE goes through the same process as LTE UE where to camp on a cell, it goes through frequency and timing synchronization to obtain the center carrier frequency as well as the allocated slot and frame timing used for the cell acquisition. In general, if MIB and SIB are properly decoded, cell ID, a subframe number, scheduling information, and system bandwidth can be detected successfully. In NB-IoT, the low complexity of devices may lead to poor synchronization and cell acquisition performance, especially due to carrier frequency offsets and poor channel estimation capacity. The following are the papers that have proposed different solutions to optimize the initial cell acquisition and initial synchronization procedure.

In [49], the authors presented a Maximum-Likelihood (ML) NPSS detector which is based on frequency domain cross-correlation metrics by using an overlap-save method. Their method achieves an average timing synchronization latency of 140 ms for the in-band deployed mode with SNR of −12.6 dB. Their proposed method showed a 34% reduction of the energy that is required for NPSS detection. However, their work showed only how much energy could be reduced with respect to the autocorrelation NPSS detection methods. It could be better to show how much of the total device’s energy is consumed by their proposed computationally complex detector, i.e., it could be more realistic to include analysis in terms of reduction with respect to the energy consumed during time synchronization but also in terms of energy optimization over the total device consumption.

In [50], the authors presented an algorithm for initial synchronization and cell search. The proposed algorithm uses NPSS for timing acquisition and initial Carrier Frequency Offset (CFO) estimation called the two-stage time domain NPSS correlation. They also used NSSS sequences for the cell ID and frame timing. Their proposed algorithm showed that under extremely low SNR and different fading conditions, NB-IoT could provide the required performance and could also quickly camp on the cell, if any. However, practical experiments are still needed to prove the feasibility of these simulations especially on how the newly introduced NB-IoT power class and actual channel variations could have an impact on the detected SNR at the base station.

In [51], the authors presented an NB-IoT framework by using an advanced signal waveform called non-orthogonal spectral efficient frequency division multiplexing (SEFDM). This waveform uses less bandwidth as compared to OFDM waveform. The designed signal could improve the data rate without the need for more bandwidth. At the base station, the minimum Euclidian norm search detector is used for better error correction. The simulation results reveal that the proposed advanced signal waveform could achieve 25% improvement on data rate as compared to the OFDM signal waveform. The work also proposed an overlapped sphere decoding (OSD) detector which reduces the computation complexity as compared to the single sphere decoding detector while guaranteeing the needed performance. However, the model does not explain the impact of CFO due to the non-orthogonality of the subcarriers on the received signal.

In [52], the work investigated the downlink synchronization signal design and proposed the novel general synchronization signal structure with a couple of Zadoff-Chu (ZC) conjugated sequences in order to remove the potential timing errors caused by large frequency offsets. Their new synchronization signal structure demonstrated better functionality with the frequency offset tolerance of up to 40 kHz. However, the model does not explain the number of samples per symbol involved in synchronization operation and decision.

In [53], the random access preamble is discussed based on the design of NPRACH for single-tone frequency hopping only. It introduces the new single-tone frequency hopping random access signal used by NPRACH in NB-IoT systems. It further explains the design rationale and proposes some possible receiver algorithms for NPRACH detection and ToA estimation. The simulation results show that NPRACH performance is improved. However, the paper did not discuss the impact of massive interference which may result in lower received Signal Interference plus Noise Ratio (SINR) at the base station such that the lower the SINR the lower the detection probability of NPRACH. In addition to that the higher the number of devices the higher probability of NPRACH preamble collision. So lower SINR detected at base station and higher collision probability both affect NPRACH detection negatively.

In [54], the authors described a NPRACH design as specified in 3GPP in standard in Release 13. They proposed a receiver algorithm for the NPRACH timing advance estimation as well as detection. The simulation results for the NPRACH detection shows that if one preamble sequence is transmitted, the detection threshold should be set between 55% to 70% of the average value to satisfy the desired NPRACH performance at the lowest SNR. The results also showed that at 5 and 11 preamble sequence transmission, the detection threshold should be 50% and 35% of the average value, respectively. It is noted that increasing the detection threshold lowers the false alarm probability, which leads to an increased likelihood of misdetection.

In [55], the authors provided a mathematical model of an NB-IoT network in order to predict the optimum performance with a specific configuration of some design parameters (i.e., repetition, number of the preamble in NPRACH per second, coverage classes and intersite distance). The paper analyzes the effects of parameter choice in outdoor, indoor, and deep indoor. The work finally proposes how to choose the optimal configuration i.e., by providing the highest throughput, as well as success probability higher than minimal success probability with minimal one being of 90%. The work showed that even though the success probability has a maximum limit, it can still be altered by modifying the number of repetitions to enhance the coverage or the system capacity in terms of throughput.

In [56], the authors presented the NB-IoT frequency diversity (FD) reception for NPSS as well as NSSS. In the reception mode, the NB-IoT UE alternatively receives the NPSS and NSSS in time domain radio frame by switching the received signals transmitted in different resource blocks in the frequency domain. Their simulation results show that using the proposed FD reception could improve the detection probability by 16% more than without applying the frequency diversity. Additionally, using FD with precoding vector switching (PVC) transmit diversity, achieves 90% of physical cell ID detection (PCID) probabilities at the average SNR of 0 dB with maximum carrier offset of 70 kHz. The method also achieves 97% of PCID detection probability without consideration of frequency carrier offset.

#### 3.1.2. Random Access Procedure

Like in LTE, NB-IoT random access (RA) is intended for initial UE uplink synchronization through which the UE acquires its unique UE ID used for communication with the base station. RA is also used to regain the lost UE access due to the long state of inactivity which has led to the loss in uplink synchronization. In NB-IoT, RA faces several challenges as seen on the research discussions; some solutions to improve the RA performance have been proposed as described in what follows.

In [57], the authors presented the random access procedure (RAP) model and analyzed the system performance by taking into consideration the configurable signal propagation model, a number of supported users per cell, and the RAP configuration parameters. The paper used the contention-based random access with Msg3 collisions instead of Msg1 collision (as multipath transmission) for the random access procedure. The proposed model results show the impact of the parameters (Msg3 transmission mode, Msg3 modulation and coding scheme (MCS), power control schemes and power ramping step) in the single-tone and multi-tone transmissions Bit Error Rate (BER) performance. The results are presented in terms of the total number of preamble transmission success, preamble retransmissions and lost preamble attempts. The work concludes that Msg3 must be considered in the random access procedure analysis, the transmission mode as well as MCS, and for better system performance and fairness distribution of UEs in the cell, it is better to configure power control correctly.

In [58], the authors analyze NB-IoT transmission delay as well as mathematical evaluation of the probability of success for the random access procedure preamble transmission. The analysis is based on three scenarios; scenario one uses minimum values of parameters, scenario two uses the intermediate values, and scenario three uses maximum parameter values. The used parameters are NPRACH periodicity, start time, number of repetitions, number of preamble attempts, and random access response window size. The average delay analysis was performed such that *k* preamble sequences are mapped in *n* subcarriers. The preamble collision occurs when multiple UEs send preamble sequences in the same subcarrier. A successful preamble attempt occurs when only one UE sends the preamble to a given subcarrier.

In [59], the authors investigated a random access optimization algorithm and summarized the NPRACH feature and hence designed random access with differentiated barring (RADB) for NB-IoT system. It is observed that the RADB could solve the preamble request conflict caused by massive NB-IoT UEs and hence provide reliable random access for latency-sensitive devices. However, the authors did not consider the problems of channel resource distribution and resource use rate.

In [60], the authors designed a new frequency hopping pattern of NPRACH preamble which uses all feasible hopping distances for a given number of subcarriers. It is seen that their proposed pattern was compatible with standards that is keeping the same NPRACH structure with only very small changes (hopping in the standard is allowed only between the subcarriers of the same resource group). Their simulation where they adopted their first traffic model which deploys 3000 devices, 48 ms and 40 ms of NPRACH preamble and periodicity, respectively, show that the proposed hopping pattern could improve the ToA estimation without additional system overhead.

#### 3.1.3. Channel Estimation and Error Correction

Like in LTE systems, NB-IoT system performance depends to some extent on the quality of the channel estimation. However, for NB-IoT systems massive deployment, the poor quality of channel estimates is highly influenced by the low complexity of the UEs that can lead to misdetection of some signals, frequency offset, phase noise, passive intermodulation (PIM) on the device level, etc. To address the challenges that affect the channel estimation as well as to improve the quality of error correction to ensure the required performance with the low complexity, several works have proposed some solutions, as summarized in the following paragraphs.

In [61], the authors presented an NPSS detection method whose timing metric is composed of symbol-wise autocorrelation and a dedicated normalization factor in an in-band downlink NB-IoT system. The authors proposed a novel low-power algorithm for frequency tracking by the use of more pilot signals as compared to the LTE system. Their algorithm is implemented to compensate for the accumulated frequency offset during the NB-IoT transmission of NB-IoT. Their proposed frequency tracking algorithm delivers high estimation efficiency in terms of Minimum Mean Square Error (MMSE), the probability of correct cell acquisition, etc. However, their study did not elaborate on what could be the impact of mobility and inter-RACT support in the cell search procedure for NB-IoT.

In [62], the authors presented a practical coverage test on the ocean; it is shown that the proposed solution (where the base station decides whether the compensated round trip delay is short or long enough to decode the preamble sequence) was done by considering NPRACH design and hence the authors proposed their solution which considers the TA adjustment. Their proposed solution proved that NB-IoT coverage could reach as far as 35 km. However, the paper does not elaborate on the solution feasibility in environments without line of sight.

In [63], the work provided optimization cases for NB-IoT downlink in terms of MCS. The work also provided the optimization cases of coverage level (CL) by taking into consideration the RACH success rate with different driving speeds of NB-IoT devices in a commercially deployed network. Their results show that the base station paging success rate is decreased as the adjacent cell interference increases. However, the decrease in MCS improves paging performance. Coverage level 0 is the best choice for NB-IoT use cases that involve mobility, whereas coverage level 1 and 2 are mostly for fixed location NB-IoT use cases.

In [64], the author presented an iterative algorithm for NB-IoT transmission procedure. The simulation results in terms of BER and blocks error rate (BLER) show that by use of concatenated error correcting codes or cryptographic redundancy and error correcting code, the algorithm improves the NB-IoT coverage and reduces the overall NB-IoT power consumption. The modification of additional correction of low reliable bits could demonstrate the error correction of the damaged messages by the noisy transmission and hence can reduce the repetition number. However, this work did not discuss how effective the algorithm is when taking into consideration different channel conditions, payload sizes, as well as different repetition numbers with respect to device signal quality.

In [67], the authors considered the presence of random phase noise of the received signals mainly caused by oscillators impairments in both the transmitting and receiving sides and how to lower the mean square error (MSE) estimates. They presented the sequential MMSE channel estimation method that could be implemented in NB-IoT systems. Their model shows that if random phase noise is considered during channel estimation, it is possible to improve the detected SNR by up to 1 dB. However, the model is assumed to be uniformly distributed hence does not present the real-time channel which is randomly changing over time.

#### 3.1.4. Co-Channel Interference

NB-IoT being deployed in the existing LTE spectrum, co-channel interference may occur between NB-IoT and LTE UEs. This is due to several reasons such as sampling rate mismatch, inter-PRB interference due to power leaking between NB-IoT and LTE PRBs, etc. To mitigate the impact of co-channel interference in the NB-IoT/LTE coexistence scenario, the following works have addressed the problems and proposed potential solutions.

In [65], the authors proposed the design guidance for channel equalization that can be used for 5G networks. The proposal set some assumptions such that the currently most used algorithms in cyclic prefix—OFDM system for pilot design, channel estimation, equalization, synchronization, and system performance analysis may no longer be applicable to NB-IoT systems. Their mathematical modeling demonstrated that channel equalization coefficients for NB-IoT UE are a set of phase-shifted CFR combination and not a simple Fourier Transforms of the channel impulse responses. This is the consequence of sampling rate mismatch between NB-IoT user and base station.

In [66], the authors established a comprehensive system model for in-band and guard-band NB-IoT by considering sample duration. They derived the mathematical expressions of received LTE and NB-IoT signals and analyzed the close-form interference power on LTE signal from adjacent NB-IoT signal. It is observed that the sample duration of NB-IoT significantly impacts the desired signal as well as interference on LTE UE; this is due to mismatched sampling rate between NB-IoT UE and the base station. Their proposed system model and derivations match the simulations, hence can be used for coexistence analysis for NB-IoT system.

**Summary:** This subsection has addressed the state of the art of NB-IoT PHY layer protocol. The main focus was set on different approaches to improve cell acquisition process, random access process, channel estimation, and interference mitigation. The next subsection focuses on MAC layer features by addressing the corresponding challenges and potential solutions.

### 3.2. Media Access Control Layer

Handling retransmissions (HARQ), multiplexing, random access, timing advance, choice of transport block formats, priority management, and scheduling are the tasks executed by the MAC layer. The discussion on this part focuses on features such as radio resource management, link adaptation, coverage, and capacity improvement, power, and energy consumption reduction, as summarized in Table 3.

#### 3.2.1. Radio Resource Allocation

In NB-IoT, resource allocation is the key feature to ensure the expected massive connections in a cell. Tone allocations, PRBs, repetition number options, power configurations, subframes, or time slots, etc. must be optimized to maximize performance with minimum possible resources. Since NB-IoT is intended for low rate, less frequent time insensitive applications but with the required performance metrics, better radio resource management will ensure the optimal resource usage for expected throughput, spectral efficiency, and coverage enhancement.

In [68], the authors discussed the impact of interference for partial deployment of NB-IoT such that if one PRB is used for NB-IoT in some of the cells, that same PRB could be used for LTE in other cells for the in-band mode of NB-IoT operation. In such a deployment, possible co-channel interference may appear between NB-IoT UE and LTE UE. The authors modeled the partial deployment in percentile such that 100%, 75%, 50%, 25% represent the percentage of cells where NB-IoT enabled. Their results were analyzed by means of cumulative density functions (CDF) of respective SINR detected and maximum coupling loss achieved. The work demonstrates possible NB-IoT interference between NB-IoT and another NB-IoT UEs from adjacent cells and between NB-IoT and LTE from the adjacent cell. The simulation is performed for the in-band mode of operation where both NB-IoT and LTE UEs share the same PRB. They proposed the PRB blanking i.e., blanking the resources that are used by NB-IoT to not be used by LTE, not even being used for CRS. Blanking of these resources will omit the interference from LTE UEs and will result in NB-IoT only access to this PRB. However, the paper did not consider the performance degradation due to reduced available radio resources after when resource blanking is applied.

In [69], the authors formulated an analytical model to characterize the maximum achievable data rate, then investigated the impact of intercell interference in a multicell environment (for in-band and standalone scenarios), and finally proposed an iterative algorithm which uses cooperative approach which takes into consideration the overhead of control channels, repetition number, intercell interference as well as time offset. The proposed sub-optimal solution ensured better radio resource allocation, which raised the data rate by 8% and reduced the overall device energy consumption by 17% with respect to the non-cooperative approach.

In [82], the authors presented preliminary results of RSSI and detected SNR by developing a DORM (integrateD cOmpact naRrowband platforM) node which was deployed on a university campus to test its practical feasibility in different indoor scenarios. Their SNR and RSSI values were observed to be in the range of 18 dB to 23 dB and −65 dBm to −70 dBm, respectively, which shows its suitability for indoor coverage. The RSSI and SNR values variations are considered to be due to different elevations that the nodes are, with respect to the serving base station. However, the paper does not explain the channel estimation and measurements quality and their impact on the achievable throughput, moreover their paper does not cover the outdoor deployment and the impact of repetition on the overall devices’ energy consumption when devices are located in different indoor environments.

In [70], the authors introduced the NB-IoT radio access strategy in detail and studied the NB-IoT scheduling problem. Their primary objective is to lower the number of used radio resource while each device’s data requirement can still be satisfied. They furthermore formulated the NB-IoT scheduling problem and proposed an efficient algorithm to overcome such a problem. Their simulation results show that they could minimize the number of NPDCCH periods (NPs) used to satisfy each device’s data requirement.

However, the repetition number is given according to the distance between the base station and the device. It could be better to use real-time channel parameters or MCS or BLER value to schedule the respective downlink channels to the devices. This is because, within the same distances, devices may experience different signal attenuation due to different factors such as fading, non-line of sight, line of sight, indoor placement, outdoor placement, underground placement, etc. Therefore, there is still an open space for practical deployment to analyze the effectiveness of the different downlink and NB-IoT scheduling schemes which considers the device’s simplicity, modulation schemes, channel conditions, and delay requirement for specific use cases.

In [71], the paper proposed a new resource allocation technique by extending the paging resource that will be specific for paging traffic offload. The authors noted that the new paging PRB could lower power consumption which is mostly used to load and offload the paging load. Also, the work proposed the selection scheme based on UE identity (ID) that is used to balance the load between the paging resource blocks. The simulation results show that power consumption reduction and resource optimal usage are of 80% and 30.5%, respectively. This work considered adding other PRBs for paging monitoring; however, the authors do not demonstrate the trade-off between the newly introduced scheme and the UE complexity requirements.

In [83], the authors proposed an enhanced access reservation protocol (ARP) that allows the device to transmit a fraction of a preamble sequence by providing an analytical model that captures the performance of ARP in terms of the false alarm, misdetection, and collision probabilities. They mathematically analyze the trade-off between the misdetection and the collision probabilities. The drawback of this protocol is that with massive NB-IoT deployment, altering the configuration of the protocol may result in detection performance degradation which can lead to huge packet loss.

In [72], the authors analyzed the impact of interference when the 15 kHz LTE system coexists with a guard-band NB-IoT system with 3.75 kHz subcarrier separation. Their simulation results demonstrated that it is desirable that the scheduler of the LTE system empties the neighboring RBs of the NB-IoT system and allocates resources if possible. The authors then proposed an NB-IoT scheduling method for the LTE system to improve the performance of the studied NB-IoT system. Their results showed that if emptying is not done, at $10^{3}$ BER there is 1 dB drop of SNR as compared to when emptying of RBs is done.

#### 3.2.2. Link Adaptation

Like in LTE, NB-IoT link adaptation involves adaptive modulation and coding schemes as well as adaptive power allocation. However, the modulation schemes are limited to QPSK to enable low complexity and hence reduce the overall power consumption. To extend the coverage and increase the link reliability, a repetition number of up to 128 times is introduced. In the literature, it is seen that NB-IoT link adaptation has several issues; potential solutions are also proposed, as summarized below.

In [31], the author formulated the scheduling issue such that the resource assignment must be in a specific format taking into an account reserved signaling resources and capabilities of the NB-IoT UE. They proposed a solution that incorporates two parameters which are (i) offset index selection (k0) and (ii) UE specific and common search space configuration. The offset index selection was chosen because with the limited k0 and varying size of payloads, it is critical to adapt the scheduling process for high resource use to accommodate more devices at the same time. Additionally, UE specific and common search space configuration were chosen because it decides the timing of NPDCCH and NPDSCH for different UEs, hence it can consequently improve the overall scheduling efficiency.

In [73], the authors presented a basic NB-IoT scheduler for NB-IoT system and analyzed the enhancements on average delay, optimal resource usage, and processing time. The proposed algorithm demonstrated that shorter NPDCCH period selection may reduce the UE average delay and optimize the overall system resource usage. Also, the model shows that the scheduling delay (k0) should be determined before the allocation of subcarriers. However, the model does not elaborate the type of configuration used since the choice of configuration such as single tone or multi-tones have a direct impact on periodicity and transmission delay and hence can directly impact the system performance.

In [74], the authors analyzed NB-IoT repetition number and bandwidth allocation and proposed analytic expressions based on SNR, bandwidth, and energy per bit that can be derived from Shannon theorem in order to characterize the impact of the repetition number as well as bandwidth allocation to different UEs. Additionally, their work proposed an algorithm for link adaptation. The algorithm exploits resource unit number, repetition as well as bandwidth. Their results show that reducing bandwidth and performing repetitions could enhance the coverage. However, the work did not consider the actual impact of channel parameters as well as NB-IoT UE impairments such as CFO which may lead to transmission errors.

In [75], the authors proposed a new NB-IoT link adaptation scheme with the consideration of the repetition factor. They claim that their proposed two-dimensional scheme is composed of Inner Loop Link Adaptation that copes with BLER by periodically adjusting the repetition number and outer loop link adaptation which coordinates the MCS and repetition number. This is because 20 dB coverage enhancement beyond LTE can be achieved by the repetition of transmitted data. So, in this work, they proposed an algorithm that dynamically chooses MCS and repetition number based on estimated real-time channel state information (CSI). However, their algorithm does not elaborate on the different NB-IoT power classes and to which range their respective coverage could be enhanced.

#### 3.2.3. Coverage and Capacity

NB-IoT support for extended coverage of up to 164 dB of maximum coupling loss is to enable the technology to be used for cellular IoT services, especially for applications that are in hard-to-reach areas. Its narrow bandwidth and support for repetition are the key features to enable the enhanced coverage.

In [10], the authors simulated and analyzed the NB-IoT wide-area rural deployment and deep indoor urban deployment by using the network parameters of one metropolitan operator. Their work showed that NB-IoT devices could still transmit and receive data at an MCL of 167dB, which is 3 dB higher than the 3GPP’s 164 dB of MCL limit set. Furthermore, in different indoor scenarios, even with an addition of 30 dB as penetration loss, NB-IoT had better outage probabilities as compare to another LTE LPWAN technology (eMTC). For outdoor and light indoor conditions with an additional 10 dB penetration loss and an average intersite distance of 2.8 km, NB-IoT had less than 0.1% of outage probability. However, despite the varying additional penetration losses of 10 dB, 20 dB and 30 dB, their simulation does not consider the impact of features such as mobility, CFO, lower power class on the achieved MCL.

In [11], the authors showed that for the maximum number of repetitions (128 times), with 15 kHz and 3.75 kHz subcarrier spacing, coverage of up to 170.2 dB MCL and 174.2 dB MCL could be achieved, respectively. The work concluded that the evaluations show that NB-IoT could provide up to 20 dB coverage enhancement in various deployment scenarios as compared to legacy LTE. Similarly, the work did not study the impact of mobility and weak channel estimation quality to the achieved MCL.

In [76], the authors proposed two less complex scheduling schemes (compared to brute-force) that can be used NB-IoT. The first proposed scheme is called the preconfigured access scheme and the second is the joint spatial and code domain scheme. Their simulation performance results (spectrum efficiency, number of active devices as well as low collision rate achieved) by the two low complex schemes were found better when compared to the ones that can be achieved by the brute-force scheme.

In [78], the authors proposed a specific unidirectional system to study the coverage enhancement by using satellite network i.e., LEO constellation. Their proposed model with the mathematical derivations shows that NB-IoT could achieve the 20 dB more than LTE achieved MCL and could still operate according to Release 13 standards. From their results, it is seen that the packet error rate (PER) of the transmitted signal is distorted by Doppler spread. However, the model does not consider the clock synchronization between NB-IoT device and satellite, which can lead to performance degradation especially caused by the CFO or the sampling rate mismatch. Additionally, the work did not consider the maximum achievable throughput when their system is employed to comment on the effectiveness of the techniques as compared to terrestrial NB-IoT deployment.

#### 3.2.4. Power and Energy Management

The NB-IoT reduced complexity is intended to reduce the power consumption in different modes. PSM and eDRX are the implemented features dedicated to foster the long-lasting battery life.

In [79], the authors presented the NB-IoT power measurement. Their measurements were set in such a way that NB-IoT transmission consumes 716 mW when at 23 dBm with a power efficiency of 37%. DL control and data signals consume 213 mW, idle-mode-eDRx and PSM consumes 21 mW and 13 μW, respectively. In general, according to their empirical measurements, it is shown that the power consumption is 10% lower than the 3GPP estimates. During measurements, parameters such as time domain repetition, I-eDRx, and PSM were taken into consideration. To characterize each component in the proposed model, several test cases such as Tx power, UL, and DL data rates, I-eDRx, and PSM were used and all parameters except one at a time were fixed. Their results showed that the NB-IoT devices power consumption is independent of the subcarrier spacing. However, the total ON time of the devices is in many cases defining the overall battery life. As their remark, the data rates do not directly impact the power consumption, but it has a major direct impact because it defines the overall device ON time. If the transmitting interval is 1 h, the device achieves only 2.5 weeks of battery life. Increasing the duration to 24 h, the lifetime of the device increases to 12.8 years in PSM.

In [80], the authors proposed a prediction-based energy-saving mechanism to reduce energy consumption by decreasing the number of scheduling request procedures. Their proposed scheme showed that it could reduce the NB-IoT active time from 5% to 16% for the medium and bad channel quality and achieve from 10% to 34% battery saving in different scenarios as compared to 3GPP consumption simulation specifications in [43].

In [81], the authors developed a semi-Markov chain with power saving mode, idle mode, random access, and transmission mode to study the energy requirement and delay performance for NB-IoT. It is noted that for massive synchronous connections, extra power is drained in random access and transmission states due to collisions. The paper further proposes an energy optimization model based on a priori method that takes into consideration the PSM duration as well as power consumption. The results demonstrate that for optimal energy and delay requirement, it is important to set the higher RACH transmission number to accommodate more delay on the UE. However, their optimization model did not consider the power consumption during the transition of different states, because when the UE is required to perform several sessions per day, it might go through several transitions that have a significant effect on power consumption. Furthermore, the mode does not include the small data transmission scheme during RRC connection as proposed in the updated standards. However, with the introduction of the new power class in NB-IoT Release 14, there is a need for practical experiments to evaluate the new coverage classes. With lower transmit power, the SNR detected at the base station becomes lower hence the device will need to perform more repetitions to enhance coverage.

### 3.3. Upper Layers

Although the focus of this paper is mainly on the features regarding PHY an MAC layers, it is still imperative to address some enhancements, challenges, and potential solutions to the upper layers. Especially the changes that are implemented in Evolved Packet Core (EPC) by adding the Service Capability Exposure Function (SCEF) to manage both IP and non-IP data packets [84].

#### Control and User Plane Optimization

**Figure 5:**
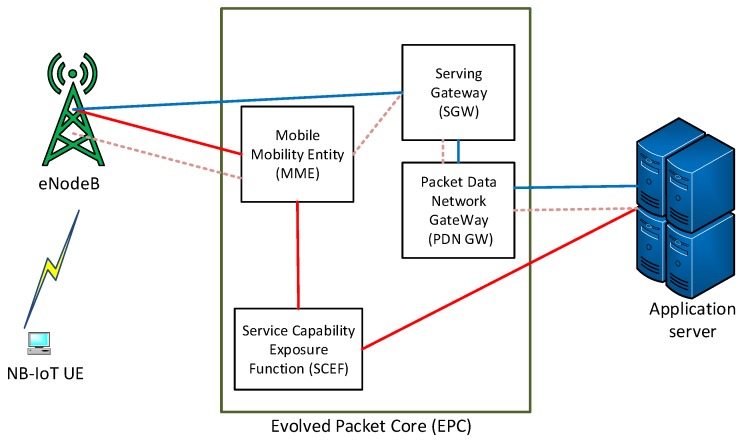
Representation of NB-IoT IP and Non-IP data path: Blue line displays the IP data path in UP mode (as Legacy LTE), Red line displays the non-IP data path in CP mode, and dashed-line displays the IP data path in CP mode.

To support massive end-to-end device connectivity with extremely low complexity and reduce the transmission signaling, NB-IoT implements new small data transmission procedures based on Cellular IoT (CIoT) Evolved Packet System on both Control Plane (CP) and User Plane (UP). These transmission procedures support small bursts of data efficiently while guaranteeing the long-range coverage as compared to legacy GPRS [85,86]. In this regard, NB-IoT is can support more than one data path in CP for the transmission of user data which is carried by the signaling messages managed by the Mobile Mobility Entity (MME) as shown in Figure 5. The procedures are optimized to efficiently support the small data transfer as follows:

- Mandatory CP CIoT EPS;
- Optional UP CIoT EPS.

CP CIoT EPS optimization encapsulates the data packets in Non-Access Stratum (NAS) by using control plane signaling messages. In this regard, this procedure is mandatory. Compared to the conventional SR procedure, the NB-IoT UE skips some steps required for each data transfer hence this optimization procedure best fits the short data transmission or reception.

On the other hand, UP CIoT EPS optimization requires the RRC connected mode to get the scheduled radio resources as well as Access Stratum (AS) between the UE and the network. This mode uses the newly introduced connection to Suspend and Resume procedures. Connection suspend procedure helps to retain the network context so that the UE can resume the connection when traffic is available. Retaining the context helps the UE and the network to skip the AS and RRC reconfiguration in each data transfer. Since it uses user plane, the UP CIoT EPS is suitable for both small and large transactions.

Furthermore, the UE in Service Request procedure (an LTE procedure used by the UE and base station to transmit or receive data in RRC idle state) is required to be in a connected state in order for base station to allocate the radio resources. For NB-IoT this SR is optional; however, NB-IoT UE that supports UP optimization needs also to support SR. For example; if the NB-IoT UE wants to transmit the uplink data in idle state, it will send the random access preamble through which the base station and UE will establish RRC connection and UE will be allocated with the radio resources for data transfer. After a certain period of inactivity, the base station initiates the release procedure.

Similarly, for UE downlink data reception, if the UE is in DRX mode, the UE regularly listens to downlink signaling and if the UE notices the paging message, it will perform the SR procedure as described in uplink data transmission. Additionally, if the UE is in PSM mode, it will be completely inaccessible until it initiates the same SR procedure for the uplink grant or by using Tracking Area Update (TAU).

There are works that are addressing the upper layers such as [77], where the authors proposed an efficient small data transmission scheme by using CP procedure. The proposed scheme enables the devices to transmit data packets through the RRC connection setup procedure when the device is in idle mode. This process reduces the signaling overhead caused by the security setup process and data radio bearer setup process. However, a suggestion could be to analyze the power consumption during this small data transmission and compare its effectiveness to when the same data is transmitted during the UP procedure.

**Summary:** This section discussed PHY layer features, highlighting the corresponding enhancements on cell acquisition procedure, random access channel estimation, and interference mitigation. It then addressed the MAC layer enhancements regarding resource allocation, link adaptation, coverage and capacity, and power management. It further addressed the upper layers changes related to cellular IoT evolved packet system optimization through user and control planes to enhance the small data packets transmissions for end-to-end massive connectivity.

## 4. Narrowband-IoT Possible Deployment Strategies

This section proposes potential deployment strategies for NB-IoT massive deployments by considering the NB-IoT support for small cells in heterogeneous network scenarios.

HetNets are effective network deployment strategies in which small cells are incorporated in macrocells with the objective of improving performance in terms of capacity, coverage, and spectral efficiency. In general, the macrocells are characterized by higher transmit power and broader range as compared to small cells. When smalls cells are overlaid in macrocells, interference becomes a concern, especially to small cell edge users. Several techniques for interference cancellation, estimation and coordination that involve frequency hopping, frequency reuse, power control etc. have been proposed; however, the performance trade-offs for the proposed techniques for macrocells and small cells are still challenging [87,88,89,90].

Similarly, NB-IoT is expected to coexist with the currently deployed legacy LTE as well as the forthcoming 5G networks. This questions the existing interference management techniques i.e., are they applicable to the newly deployed technology since NB-IoT is expected to support different power classes while maintaining the low complexity which can severely affect the channel estimation quality and hence interference estimation quality. In NB-IoT coexistence with the legacy cellular networks, the possible deployment scenarios are as follows:

- Synchronous NB-IoT deployment in all small cells;
- Asynchronous NB-IoT deployment in all small cells;
- Synchronous NB-IoT deployment in small cells and Macrocells;
- Asynchronous NB-IoT deployment in small cells and LTE in macrocells.

**Figure 6:**
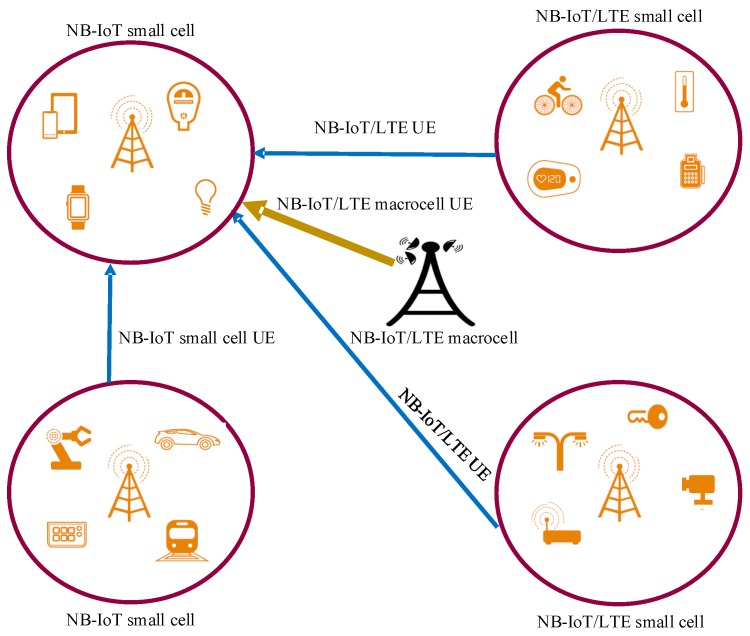
Summary of NB-IoT deployment strategies. For example, when NB-IoT is deployed in macrocell and LTE in small cell, when LTE is in macrocell and NB-IoT is in small cells, when NB-IoT is in macrocell and small cells support both NB-IoT and LTE, and when LTE is in macrocell and LTE/NB-IoT is in small cells

These scenarios, as shown in Figure 6, are detailed in what follows.

### 4.1. Synchronous NB-IoT Deployment in All Small Cells

This is the NB-IoT deployment strategy which is enabled in all the small cells by using the same physical resource blocks. All the small cells are synchronized in such a way that with the same PRBs, all the NB-IoT UEs are using the transmit power that is configured regardless of its maximum transmitting power capacity. This means that even though NB-IoT devices might support different power classes such as 14 dBm, 20 dBm, or 23 dBm, the NB-IoT devices will only be configured to use the minimum allowed transmit power in order to avoid causing the co-channel interference to other UEs using the same radio resources. In this strategy, power control may be the key feature to ensure the required performance. However, cell edge UEs may still suffer from the interference problem. This interference may highly be increased due to the low channel estimation quality of NB-IoT UEs associated by its reduced computational complexity.

### 4.2. Asynchronous NB-IoT Deployment in All Small Cells

This deployment strategy is employed in such a way that NB-IoT is enabled in all small cells by using different physical resource blocks. This implementation may avoid the interference between NB-IoT UEs from different small cells; however, this may result in co-channel interference between NB-IoT and LTE UEs that are using the same radio resources. When deploying under this strategy, it is imperative to implement proper frequency planning as well as proper power configuration for NB-IoT devices. As seen from the state of the art, some works have proposed blanking of the radio resources to the adjacent cells for the resources that are already occupied by NB-IoT even in the cells that NB-IoT is not enabled. However, blanking of the resources is a wastage of resources, so, there should be some other means such as frequency hopping to avoid wastage of resources (blanking) as well as to mitigate interference.

### 4.3. Synchronous NB-IoT Deployment in Small Cells and Macro Cells

In this strategy, NB-IoT is enabled in the small cells as well as in macro cell on the same PRBs. Macrocell UEs are configured to use higher transmit power as compared to small cell UEs while keeping the same PRBs for NB-IoT while others left for legacy LTE. Possible co-channel interference may occur in small cell edge UEs if the UEs are scheduled on the same resource units. The impact may further increase for UEs under mobility which might require the use of handover for smoothing the UEs transition from one serving cell to another. From our review, no work has addressed the interference cancellation mechanism for such a case. It is imperative to employ the existing geographical planning, frequency reuse, frequency hopping, and power control while considering the low complexity but high coverage range NB-IoT.

### 4.4. Asynchronous NB-IoT Deployment in Small Cells and LTE in Macrocells

In this strategy, NB-IoT uses separate PRBs between small cells and macro cells. This means that one or more PRBs are used for small cells and different PRB(s) for the macrocells. If the PRBs are not well planned, NB-IoT users from adjacent cells (using the same resource units) may suffer from interference. Also, LTE users that are using the same resource elements may interfere with small cell or macro cell UEs. Different transmit power control configurations may be used to control interference.

The choice of the deployment strategy depends on several factors such as use-case requirements, environmental conditions, equipment quality, etc. It is imperative to implement better interference estimation, mitigation or management techniques that will ensure better performance and spectral efficiency for the massive NB-IoT deployment in coexistence with other technologies.

**Summary:** This section has presented the possible NB-IoT deployment strategies by considering the NB-IoT support for small cells in coexistence with legacy LTE in HetNet scenario.

The following section presents the open research challenges to motivate future research directions.

## 5. Open Research Questions and Discussion

### 5.1. Battery Life

PSM and eDRx were introduced in NB-IoT Release 12 and 13 to lengthen the NB-IoT devices’ battery life. Moreover, the most recent updates require the UE to be able to transmit during RRC-idle mode which will reduce the required ON time for data transmission. However, devices experiencing bad channel conditions due to hard-to-reach areas will require to perform several retransmissions per session, which will drain the device’s energy and hence shortens the battery life. Similarly, devices that require a relatively large number of reporting sessions per day will consume more energy, which makes energy management a concern. As seen in Section 3, most of the proposed algorithms are power hungry because most of the power is consumed during transmission and reception. Therefore, energy harvesting alternatives such as solar, biogas, vibrations, etc. that will lengthen the NB-IoT device battery life should be introduced to complement or replace frequent battery charging.

### 5.2. Radio Resource Management

#### 5.2.1. Tones Allocation

As seen in the literature, most of the articles consider single-tone allocation for the simplicity in the simulation, thus, multi-tone allocation is not well studied. This causes a knowledge gap in the effectiveness of different tone-allocation possibilities. Moreover, for guard-band, in-band and standalone it is still not clear about the respective performance metrics that could be achieved in terms of throughput, coverage range, interference robustness etc. This restricts to a certain extent the optimal choice of deployment for a large number of devices with the required performance. Furthermore, different frame structures, especially for TDD configurations, are not discussed even though NB-IoT is required to support TDD. Therefore, optimal resource use techniques must be proposed that incorporate repetition, mobility, tones allocation, etc. for efficient spectrum usage.

#### 5.2.2. Interference Mitigation

Interference prediction, estimation, cancellation, and coordination techniques for NB-IoT become a challenge. This is because of the sharing of spectrum resources between NB-IoT and legacy LTE. Similarly, with NB-IoT being deployed in a small cell or macrocell scenarios in heterogeneous networks, interference becomes a concern. Several works have tried to address this by means of resource blanking, power control, or better uplink and downlink scheduling schemes and frequency and timing synchronization, etc. However, it is still challenging to incorporate the NB-IoT features such as repetition, low complexity (which affects channel estimation quality), and mobility in deploying the already existing LTE interference management techniques. As seen in the possible NB-IoT deployment scenarios above, there is still a need for deploying effective schemes that will ensure better NB-IoT performance without degrading the LTE performance [91,92].

### 5.3. Mobility Management

As seen in Section 2, most of the simulation works have ignored the mobility impact of NB-IoT channel modeling. However, for use cases that involve movement, Doppler shift has to be taken into consideration during channel estimation, which might slightly increase the device complexity to support handover and other mobility features such as the support for inter-RAT mobility during idle mode [93,94]. The increase in NB-IoT UEs mobility makes the channel suffer from fast varying channel conditions, due to which adaptive transmission schemes that might involve channel estimation, error correction, etc. must be implemented.

Therefore, applying intelligent/adaptive algorithms that are low power and optimal for repetition number, yet mobility-aware, is of great importance. The algorithms could involve low-power frequent CSI reporting, early data transmission by using both user and control plane in either Msg 3 or Msg 4.

### 5.4. Latency

NB-IoT latency tolerance is set to 10 ms. This is due to its support for use cases of UEs that are in environments with bad channel conditions [95,96,97]. Initial cell acquisition, frequency, and timing requirements, RACH transmission, half duplex mode of transmission and several repetitions that are performed during transmission are some of the features that play part in the overall data transmission delay. Several works are trying to reduce the timing requirement so as to reduce transmission latency of devices; however, most of the works have not addressed delay by taking into consideration the massive congestion that is expected for the IoT networks, processing delays due to low complex devices, queuing delays, propagation delays especially with long-range feature, as well as errors and error recovery.

However, early data transmission schemes and the second NB-IoT HARQ process for devices that have good channel conditions are among the features that can be used to reduce the transmission latency and improving the transmission link performance. However, only a handful of research articles have discussed the effectiveness of these processes when applied in NB-IoT.

### 5.5. Semi-Persistent vs. Dynamic Scheduling

Most of the NB-IoT literature addresses dynamic uplink and downlink scheduling by studying the scheduling of logical channels and signals. There are still very few NB-IoT studies about the effectiveness of Semi-Persistent Scheduling schemes (SPS) even though SPS helps to reduce the NPDCCH overhead as compared to dynamic scheduling. It provides the NB-IoT UEs with longer allocated resources (more than one subframe) so that the NB-IoT device will not need the frequent downlink assignment as well as an uplink grant which is delivered by NPDDCH for each subframe. However, for applications that involve mobility or fast varying channel conditions, how is this scheduling scheme going to be effective knowing that NB-IoT has poor channel estimation capabilities as compared to LTE?

### 5.6. Random Access

Massive NB-IoT modules that try to request the radio channel resources at the same time for uplink data transmission may suffer from random access preamble collision. This is caused by several factors such as detection inaccuracy that may not satisfy the detection threshold, the high probability of false alarm, etc. Several works have proposed random access preamble detection algorithms (i.e., random access with differential barring etc.) and others have developed mathematical models to characterize the preamble transmissions in order to improve the NPRACH success rate and better time-of-arrival estimation and other NPRACH performance improvements. However, it is still unclear which scheme is effective for massive deployment, since most of the proposed schemes do not consider the heterogeneous network architecture, channel estimation impairments, or realistic channel conditions [98,99].

### 5.7. Timing Advance (TA)

When the base station responds to NB-IoT UEs about RRC connection request, it incorporates the TA command to be used for NB-IoT UE terminal data uplink transmission timing (i.e., to time-synchronize the UEs to the base station and help to compensate the propagation delays). However, for NB-IoT UE, the TA adjustment accuracy of the signaled timing advance with respect to the prior uplink transmission may highly be affected by the massive number of NB-IoT devices contending for the access. This is because the base station may need to correct some UE timing while for other NB-IoT UEs that had already transmitted NPRACH could receive the random access response which is not intended for them. Some works have addressed the receiver algorithms for NPRACH TA estimation as well as detection timing advance adjustment decoding schemes to improve the estimation but the NB-IoT receiver sensitivity and weak channel estimation quality still negatively affect the TA adjustment.

### 5.8. Cell Search and Initial Synchronization

NPSS and NSSS are two signals based on frequency domain Zadoff-Chu sequence that are used for NB-IoT time and frequency synchronization to the base station. According to NB-IoT standard, NPSS and NSSS may not be transmitted on the same antenna port hence NB-IoT initial synchronization may rely on NPSS only. The challenge is that the imperfect channel conditions may severely affect the cell camping procedure as a small CFO may result in a phase shift to a received frequency domain sequence which as a consequence may degrade the cell search and synchronization performance. To improve this, frequency diversity techniques should also be used for NPSS and NSSS reception improvement.

### 5.9. Unified NB-IoT Testing Tool

Since NB-IoT is a promising technology, there should be a unified testing tool used as a reference to verify if the produced products comply with the standards. Taking Bluetooth as an example, for better compatibility towards different available products from handsets to car kits, Profile Tuning Suite (PTS) software is used to automate the compliance testing to specific Bluetooth function. So, to support compliance with standards and hence backward compatibility and interoperability, what is the testing tool to validate if different available products will fit standards? Similarly, for simulation purposes, most of the works choose the parameters that can generate results easily. If there is a concrete simulation model that takes into account the major NB-IoT features and incorporating all the possibilities from repetition number allocation, mobility selection, modulation and coding scheme, real-time channel variations, etc. it would be easier to get realistic modeling for different scenarios.

### 5.10. Backward Compatibility and Interoperability

A ten-year telecommunication generation is characterized by different changes in releases and updates. In order to reach their lifespan as compared to what the standards stipulate, NB-IoT devices should operate for around ten years with a single battery charge. Whenever new releases or updates are introduced, backward compatibility and interoperability should be possible. Apparently, the device complexity is set as low as possible; will these simple devices (hardware) support hard and robust algorithms that will be implemented by over-the-air upgrades/updates to satisfy the demands of future NB-IoT use cases?

**Summary:** This section has presented the open research questions regarding battery life, radio resource allocation, cell search, and initial acquisition procedures, mobility management, latency, random access, etc., as summarized in Table 4, in order to motivate future research directions. The next section concludes the paper.

## 6. Conclusions

Due to the fact that most of the existing works are segmented and only consider one or two releases in their corresponding studies or simulations, this paper has presented a comprehensive overview of NB-IoT standard from Release 13 to Release 16 prospects to enhance and enable more realistic research. It further presented the detailed current state of the art of NB-IoT based on the ongoing discussion on NB-IoT protocol stack along with the related contributions and analyzed the knowledge gaps by using NB-IoT standard as a benchmark. It is observed that most of the articles focus on improving one or few features while neglecting others, it could be better to display the trade-offs between the improvement feature and the neglected ones, i.e., performance trade-off between PHY and MAC layer when one feature is changed in either of the layers, the impact of repetition on overall energy consumption, CFO on channel estimation quality etc. This paper also presented the NB-IoT deployment strategies to highlight the coexistence possibilities with other legacy technologies i.e., LTE, by considering the NB-IoT support for small cells in HetNet scenarios. Lastly, it discussed the open research challenges and the future common research focus on NB-IoT i.e., battery life, optimal resource usage, handover support during mobility, transmission latency, scheduling, etc. To the best of the author’s knowledge, this is the first survey that covers broadly these mentioned contributions and hence this work will help the researchers get most of the needed information to accelerate their research by finding the relevant information and sources for deeper exploration of the research concepts as well as finding possible solutions.

## Figures and Tables

**Table 1:** Summarized comparison of this survey’s contribution with respect to the existing surveys.

Survey	The Third Generation Partnership Project				Layers		Deployment Strategies
**[Ref]**	**Rel 13**	**Rel 14**	**Rel 15**	**Rel 16**	**Physical**	**Media Access Control**	
[28] 2017	√						
[29] 2017	√	√					
[30] 2017		√					
[31] 2017	√						
[32] 2018	√						
[33] 2019	√	√					
This survey	√	√	√	√	√	√	√

**Table 2:** Articles on the proposed PHY layer enhancement techniques.

Feature	Article	Technique Used	Enhancement Criteria	Limitation
Cell Acquisition	[49]	Maximum-Likelihood (ML) NPSS detector	Average latency reduction for timing synchronization	It is a computationally complex detection method
	[50]	Cell search and initial synchronization algorithm	Time and frequency synchronization by using NPSS and NSSS with two-stage time domain NPSS correlation	mobility and new NB-IoT transmit power are not considered which have a direct impact on inter-RAT camping and the detected SNR, respectively
	[51]	Non-orthogonal spectral efficient frequency division multiplexing (SEFDM) waveform and an overlapped sphere decoding (OSD) detector	Resource optimization by the use of less bandwidth with better data rates compared to OFDM signal waveform	The proposed method would lead to sampling rate mismatch, carrier frequency offset and also will need to raise the computation complexity to NB-IoT UE
	[52]	New synchronization signal structure with Zadoff-Chu conjugates	Minimization of timing errors due to low-complexity NB-IoT frequency offset	If the same model is used for uplink synchronization it might lead to estimation errors if mobility is involved in NB-IoT
	[53]	NPRACH detection and time-of-arrival estimation for NB-IoT system	Enhancement on cell acquisition and channel estimation accuracy	The algorithm might not work for multi-tone allocation. Also, frequency hopping may raise power consumption as well as device complexity
	[54]	Receiver algorithm for NPRACH timing advance estimation and detection	Modeling the detection threshold to satisfy the NPRACH performance by lowering the probabilities of false alarm	The paper did not explain how receiver sensitivity can affect the NPRACH detection
	[55]	Mathematical modeling of NB-IoT performance	Throughput enhancement and NPRACH optimization by the use of repetition number, NPRACH preamble transmission per second and intersite distance	The work did not include some parameters such as the impact of mobility and how the achieved MCL for different coverage classes can impact the repetition assignment
	[56]	NPSS and NSSS frequency diversity reception	Time and frequency synchronization for cell search improvement	Alternative switching of NPSS and NSSS may require additional control commands which may lead to higher device complexity
Random Access	[57]	Configurable signal propagation model	System performance analysis in terms of number of supported devices, BER performance, preamble retransmissions, etc.	The impact of preamble retransmission on the overall transmission latency is not considered
	[58]	Mathematical evaluation of RACH preamble transmission	Analysis of NB-IoT transmission delay by using periodicity, start time, number of repetitions, number of preamble attempts and random access response window	Their model used minimum, intermediate, and maximum values for simulation which is so deterministic. However, it could be better to use random distribution to characterize NB-IoT realistic channel variations
	[59]	Random Access with differentiated barring (RADB) algorithm	Minimization of random access collision	Not resource efficient method since it does not include the impact of scheduling in different tone configurations
	[60]	New frequency hopping pattern of NPRACH preamble	Time-of-arrival estimation by the use of all the hopping distances	It only used a small cell scenario, if applied in dense NB-IoT network, estimation by considering all hopping distances may lead to system overhead and possible interference
Channel estimation	[61]	Frequency tracking algorithm	Frequency synchronization, as well as channel estimation for NB-IoT systems	More pilot signals, are used. This increases the overhead and hence can degrade the spectral efficiency
	[62]	Timing advance (TA) adjustment	Preamble sequence decoding by means of round trip estimation for coverage enhancements (on the sea)	It might not work for applications that do not involve a direct line of sight such as in dense urban environment
	[63]	MCS and coverage level optimization	Mobility effect on different coverage levels and how MCS affect paging performance	The channel model does not include other factors such as the effect of repetition, multipath, different Tx power for NB-IoT UEs as well as carrier frequency offset and inter-RAT operability
	[64]	New iterative algorithm for NB-IoT transmission scheme	NB-IoT error correction by using cryptographic redundancy and error correcting code	The channel estimation model to characterize NB-IoT transmission is not good, because some errors might be due to intersymbol interference and others due to intercarrier interference however the model does not explain
Interference mitigation	[65]	Channel Equalization algorithm	Intersymbol Interference mitigation by the phase-shifted channel frequency responses (CFR) to conquer the sampling mismatch between NB-IoT and base station	The proposed model did not consider the NPSS and NSSS impact ON time and frequency synchronization
	[66]	Mathematical model for sample duration in LTE and NB-IoT system	Interference and close-form interference analysis due to sampling mismatch between NB-IoT and base station	The model is computational complex when implemented in NB-IoT systems

**Table 3:** Articles on proposed MAC layer enhancement techniques.

Feature	Article	Technique Used	Enhancement	Limitation
Resource allocation	[68]	Resource blanking	Interference cancellation by resource blanking	The proposed technique may lead to performance degradation in terms of spectral efficiency, especially for NB-IoT massive deployment.
	[69]	Iterative algorithm by a cooperative approach	Radio resource management in terms of scheduling index, repetition number and interference	The proposed solution is sub-optimal hence it does not provide maximum achievable performance in terms of maximum rate and capacity
	[70]	Scheduling algorithm	Efficient resource allocation by reducing the NPDCCH periods	Mobility is not considered and reducing NPDCCH period could lower the channel estimation quality hence may degrade the performance by unrealistic channel estimation
	[71]	Resource allocation technique by extending the specific PRB for paging traffic offload	power consumption reduction for NB-IoT UE during paging loading and offloading	The use of specific PRB for paging offloading is not an efficient use of the existing resource blocks. Also, the model is not applicable in standalone mode.
	[72]	NB-IoT scheduling algorithm	Interference analysis for 15 kHz LTE coexistence with 3.75 kHz guard-band NB-IoT	Emptying the LTE resource is not efficient resource use. Also, the model is not applicable for the standalone mode of deployment
Link adaptation	[73]	NB-IoT basic scheduler algorithm	Optimal resource usage by considering an average device delay and processing time	The scheduler did not consider semi-persistent scheduling, especially for inter-RAT networks
	[31]	Offset index selection and UE specific and common search spaces for NB-IoT dense networks	Cell capacity enhancement by means of optimal scheduling	Did not consider the number of sessions that each device has to transmit with respect to different requirements and use cases
	[74]	Link adaptation algorithm by using the mathematical expression of Shannon theorem	Coverage enhancement by characterizing SNR, repetition number and NB-IoT supported bandwidth	The work did not consider the impact of channel state information on UE link adaptation
	[75]	Two-dimensional NB-IoT dynamic link adaptation algorithm	Optimization of repetition number by dynamically adjusting MCS to ensure better BLER and BER performance	the model does not encompass the effect of speed and the deployment of the optional HARQ process to ensure better channel modeling
Coverage and capacity	[11]		NB-IoT coverage comparisons in different scenarios for 15 kHz and 3.75 kHz spacing	The channel estimation impairments, carrier offset as well as mobility with respect to different configurations are not considered for the claimed 170 dB of achieved MCL of NB-IoT
	[76]	Preconfigured access scheme and the joint spatial and code domain scheme	capacity and spectral efficiency improvement	It can only be applicable in small cell configurations when NB-IoT is deployed in large scale, preconfiguring access for different require
	[77]	Control plane small data transmission scheme	Effective data transmission enhancement by transmitting small packets in RRC connection set up	This scheme may results in NB-IoT signaling overhead due to Radio Resource Control (RRC) connection setup process encompassed with small data
	[10]	UE coverage and capacity simulation measurement based on real operators network parameters	NB-IoT enhanced coverage measurements by the use of real network configuration parameters	Optimal repetition number for NB-IoT devices is not considered, with additional penetration loss, it does not explain the additional repetition requirement to enhance the coverage while guaranteeing the required performance
	[78]	Low Earth Orbit (LEO) satellite to extend NB-IoT coverage	NB-IoT Coverage extension beyond LTE achieved link budget	The work did not consider the impact of repetition number on extended coverage as well as time and frequency synchronization that can lead to sampling rate mismatch as well as carrier frequency offset for low-end NB-IoT modules
Power management	[79]	Practical power measurement	Power consumption analysis for NB-IoT by varying payloads and repetition numbers, I-eDRx and PSM	Using two devices is not representative massive NB-IoT devices in the because different chips have different power consumption depending on the enabled features such as inter-RAT support that can affect the overall device consumption
	[80]	Prediction-based energy-saving algorithm	Reduction of power consumption by reducing the scheduling request procedure	The solution is not optimal because it reduces scheduling request without considering the device requirement with respect to channel parameters
	[81]	Semi-Markov chain for energy evaluation	Energy consumption and delay requirement evaluation for NB-IoT systems by considering the four states, namely power saving mode, idle mode, RACH procedure, and transmission mode	The model does not include the energy consumption during transition between the four mentioned modes and it does not include the impact of repetition on the device power consumption

**Table 4:** Open Research Questions related to the physical layer, MAC layer, and standard.

Physical Layer	MAC Layer	Standard
Radio resource management	Timing advance adjustment	Support for small cell
Frequency and time synchronization	Dynamic scheduling and semi-persistent scheduling	TDD support
Random access	Latency	Antenna diversity
Channel estimation	Power management	Mobility and handover support
Error correction	Network throughput	More efficient group messages
Link adaptation	Control packet overhead	Multicarrier operation
Interference mitigation	Control plane small data transmission	Network management tool for UE differentiation
